# Effects of exercise intervention on glucose and lipid metabolism in patients with type 2 diabetes mellitus complicated by non-alcoholic fatty liver disease: a three-level meta-analysis

**DOI:** 10.3389/fnut.2026.1907058

**Published:** 2026-08-11

**Authors:** Xuan Wang, Zongqiang Jin, Yanmei Niu

**Affiliations:** 1 School of Sports Training, Tianjin University of Sport, Tianjin, China; 2 College of Medical Technology, Tianjin Medical University, Tianjin, China

**Keywords:** exercise, glucose and lipid metabolism, NAFLD, T2DM, three-level meta-analysis

## Abstract

**Objectives:**

This systematic review and three-level meta-analysis evaluated the effects of exercise interventions on glycemic control, lipid metabolism, body composition/fat distribution, and liver function in patients with type 2 diabetes mellitus (T2DM) complicated by non-alcoholic fatty liver disease (NAFLD), and examined potential sources of heterogeneity relevant to individualized exercise prescription.

**Methods:**

Randomized controlled trials were searched in Web of Science, PubMed, Cochrane Library, EBSCO, Embase, and CNKI from database inception to February 2026. The review followed PRISMA 2020 and used a three-level meta-analytic model to account for dependent effect sizes extracted from the same study. A priori subgroup analyses were conducted by exercise modality, and meta-regression was used to explore heterogeneity.

**Results:**

Twenty-four studies involving 2,578 participants were included. Overall, exercise reduced BMI (Hedges’ *g* = −0.36, *p* < 0.001), FPG (Hedges’ *g* = −0.42, *p* < 0.001), HbA1c (Hedges’ *g* = −0.52, *p* < 0.001), HOMA-IR (Hedges’ *g* = −0.48, *p* < 0.001), LDL-C (Hedges’ *g* = −0.36, *p* = 0.001), TG (Hedges’ *g* = −0.45, *p* < 0.001), ALT (Hedges’ *g* = −0.41, *p* < 0.001), AST (Hedges’ *g* = −0.38, *p* < 0.001), and GGT (Hedges’ *g* = −0.40, *p* < 0.001), and increased HDL-C (Hedges’ *g* = 0.28, *p* = 0.009). Modality-specific analyses suggested that MICT may be more favorable for FPG, LDL-C, VAT, and HFC, whereas HIIT showed larger effects for HbA1c, HOMA-IR, TG, ALT, and GGT. Heterogeneity was substantial for TG, ALT, AST, HbA1c, and GGT in the primary analyses; however, sensitivity analyses reduced *I*^2^ below 50% without materially altering the pooled effects. Evidence for resistance training was limited by the small number of studies and should be interpreted cautiously. Meta-regression suggested that medication status and exercise frequency may be potential contributors to between-study heterogeneity.

**Conclusion:**

Exercise, particularly aerobic exercise, appears to be a beneficial adjunctive strategy for improving metabolic and liver-related outcomes in patients with T2DM and NAFLD. Because clinical heterogeneity, medication use, dietary co-interventions, and study quality may influence the estimates, modality-specific conclusions should be applied cautiously and individualized according to patients’ metabolic profile, comorbidities, and exercise tolerance.

**Systematic review registration:**

https://www.crd.york.ac.uk/PROSPERO/view/CRD420261327724, identifier CRD420261327724.

## 1 Introduction

Type 2 Diabetes Mellitus (T2DM) represents one of the major chronic disease challenges worldwide. According to the latest data published by the International Diabetes Federation (IDF), the global T2DM population is projected to reach 783 million by 2045 (1). The prevalence of T2DM in China has risen to 12.4% (2). Uncontrolled T2DM can lead to cardiovascular diseases (3), cerebrovascular diseases (4), diabetic neuropathy (5), diabetic foot (6), and various other complications. Epidemiological data show that the prevalence of Non-Alcoholic Fatty Liver Disease (NAFLD) among T2DM patients ranges from 50 to 70% (7). These two conditions are mutually causal and form a vicious cycle (8). When T2DM coexists with NAFLD, patients face significantly increased risks of liver cirrhosis and hepatocellular carcinoma (9). Currently, pharmacological treatment for T2DM complicated by NAFLD may increase liver burden or cause side effects (10, 11). Therefore, exercise intervention has been recognized by the American Medical Association and the American Association for the Study of Liver Diseases as an effective non-pharmacological approach for managing both T2DM and NAFLD (12, 13).

A substantial body of research demonstrates (14, 15) that Aerobic Exercise (AE), including Moderate Intensity Continuous Training (MICT), High-intensity Interval Training (HIIT), and Resistance Training (RT), can effectively reduce patients’ body weight, blood glucose levels, and intrahepatic fat content by activating the AMPK signaling pathway, improving skeletal muscle glucose metabolism, and promoting hepatic fatty acid oxidation (16, 17). Evidence synthesized from randomized trials and meta-analyses indicates that structured exercise interventions can reduce ectopic fat accumulation in individuals with T2DM, particularly visceral adipose tissue and hepatic fat, and that these metabolic benefits may occur even without substantial weight loss (18).

Despite the established benefits of exercise intervention, significant heterogeneity remains in existing research (19). Effect sizes vary considerably across studies regarding the improvement of liver biochemical indicators, lipid profiles, and intrahepatic fat content (20). The optimal exercise modality for T2DM patients complicated with NAFLD remains unclear. Three-level meta-analysis is an advanced effect size analytical method that can distinguish between within-study and between-study heterogeneity, avoiding overestimation of statistical power (21, 22). Therefore, this study constructs a three-level meta-analysis model to evaluate the effects of exercise intervention on glucose and lipid metabolism in T2DM patients with NAFLD, exploring the effectiveness of exercise and the efficiency of different exercise modalities.

## 2 Materials and methods

### 2.1 Study design

This study strictly followed the PRISMA 2020 statement (updated version of the PRISMA guidelines for systematic reviews and meta-analyses) (23) to conduct a three-level meta-analysis. Additionally, this study was registered in the International Prospective Register of Systematic Reviews (PROSPERO, Registration Number: CRD420261327724).

### 2.2 Literature search and strategy

Computer searches were conducted across Web of Science, PubMed, Cochrane Library, EBSCO, Embase, and China National Knowledge Infrastructure (CNKI) from database inception to February 2026. Search terms were constructed according to the PICOS framework and covered five concept blocks: population (T2DM and NAFLD), intervention (exercise therapy, MICT, HIIT, RT, Combined Exercise (CE), walking, cycling, yoga, and related terms), comparison (usual care, no exercise, or maintenance of habitual activity), outcomes (glycemic, lipid, anthropometric/fat-distribution, and liver-function indicators), and study design (randomized controlled trials). The final electronic search was completed in February 2026. Title/abstract screening, full-text assessment, and final inclusion were completed before manuscript submission in June 2026. All studies included in the final analysis were peer-reviewed full-text articles and were publicly available before the predefined search cutoff. The exact screening log and database-specific strategies are provided in Supplementary Table S1.

### 2.3 Inclusion and exclusion criteria

#### 2.3.1 Inclusion criteria

(1) Population: T2DM patients complicated with NAFLD;
(2) Intervention: AE including MICT, HIIT, RT and CE (AE combined with RT);
(3) Comparison: Maintaining original exercise habits or no intervention control group;
(4) Outcomes: At least one glucose and lipid metabolism indicator;
(5) Study design: Randomized controlled trials.

#### 2.3.2 Exclusion criteria

(1) Population: Patients with only T2DM or NAFLD; animal experiments;
(2) Intervention: Intervention duration ≤ 4 weeks; nutrition intervention only without exercise intervention;
(3) Comparison: Control groups with interrupted current lifestyle and uncontrolled activities beyond specified intervention period;
(4) Outcomes: Studies reporting at least one relevant metabolic, liver-function, or hepatic fat-related outcome, including anthropometric indicators: Body mass index (BMI): Glycemic indicators: Fasting plasma glucose (FPG), Glycated hemoglobin (HbA1c), Homeostatic model assessment of insulin resistance (HOMA-IR); lipid indicators: Total cholesterol (TC), Triglycerides (TG), Low-density lipoprotein cholesterol (LDL-C) and High-density lipoprotein cholesterol (HDL-C); liver-function indicators: Alanine aminotransferase (ALT), aspartate aminotransferase (AST), Gamma-glutamyl transferase (GGT); fat-distribution indicators: intrahepatic triglyceride (IHTG), hepatic fat content (HFC), visceral adipose tissue (VAT), and fatty liver index (FLI).
(5) Study design: Systematic reviews or meta-analyses, non-peer-reviewed articles, conference abstracts, and non-Chinese and non-English literature.

### 2.4 Literature screening

EndNote 20 was used to merge search records and remove duplicates. After deduplication, records were exported to Excel (Microsoft, 2021). Wang Xuan and Niu Yanmei independently screened the titles and abstracts according to the predefined eligibility criteria and subsequently assessed potentially eligible reports in full text to determine final study inclusion. Disagreements were resolved through discussion; when consensus could not be reached, Jin Zongqiang served as the third reviewer for adjudication. For reports with incomplete numerical data, the corresponding authors were contacted where possible. Only peer-reviewed full-text articles published in Chinese or English were eligible for final inclusion.

### 2.5 Data extraction and conversion

Following study selection, Wang Xuan and Niu Yanmei independently extracted the data using a standardized Excel spreadsheet (Microsoft, 2021). Any discrepancies were resolved through discussion or, when necessary, adjudicated by Jin Zongqiang. including: (1) Basic study information (first author, publication year, etc.); (2) Participant characteristics (age, sample size, BMI, etc.); (3) Exercise modalities (MICT, HIIT, RT, CE); (4) Exercise duration, session length, and frequency; (5) Outcome indicators: The primary outcomes were IHTG, HFC, FLI, HOMA-IR, HbA1c, FPG, reflecting hepatic fat accumulation and key glucose metabolic disorders, the prominent pathological features of individuals with type 2 diabetes mellitus combined with NAFLD. Secondary outcomes contained lipid profiles, liver function biomarkers, anthropometric index and visceral fat indicator, namely TC, LDL-C, TG, HDL-C, ALT, AST, GGT, BMI and VAT.

For studies reporting standard error (SE), formula (1) was used (24): (1) $\text{SD}\,\text{SE}=\sqrt{\text{n}}$, where n is the sample size. For studies reporting 95% confidence interval (95% CI), formula (2) was used (25): $S\,D=\frac{2\times 1.96\,\text{Upper}\,95\%\,\text{CI}-\text{Lower}\,95\%\,\text{CI}}{2\times 1.96}$, where 1.96 is the critical value for 95% CI under normal distribution.

### 2.6 Risk of bias assessment

Two researchers independently assessed risk of bias using the Cochrane Risk of Bias tool (RoB2) in Review Manager 5.0, evaluating five domains: randomization process, deviations from intended interventions, measurement of outcomes, completeness of outcome data, and selective reporting. Disagreements were resolved through discussion, with a third researcher brought in if necessary.

### 2.7 Statistical methods

All statistical analyses were conducted in R 4.5.2 using the metafor package (Version 3.8-1). All statistical tests were two-tailed with significance level α = 0.05. To address the nested data structure of multiple effect sizes within included studies, a three-level meta-analysis model was constructed with three levels: Level 1 (effect size level), Level 2 (within-study level), and Level 3 (between-study level) (21). A mixed-effects model was employed using Restricted Maximum Likelihood (REML) estimation for model parameters. The model formula was: yijk = β0 + u(j) + v(jk) + e(ijk), where yijk represents the i-th effect size (Hedge’s *g*) for the k-th intervention group in the j-th study, β0 is the overall pooled effect size, u(j) is the random effect between studies, v(jk) is the random effect between groups within studies, and e(ijk) is the sampling error. Effect size (Hedge’s *g*) = [[(n1+n2)/(n1 × n2)] + g^2^/(2 × (n1+n2))]*J, where J represents the small sample correction factor, n1 is the sample size of the experimental group, n2 is the sample size of the control group.

Between-study heterogeneity was assessed using *I*^2^, with 0–25, 26–50, and > 50% interpreted as low, moderate, and high heterogeneity, respectively. Subgroup analyses by exercise intervention modality (HIIT, MICT, RT, and combined exercise) were specified a priori in the review protocol because these modalities differ in intensity pattern, dominant energy systems, weekly energy expenditure, neuromuscular stimulus, and expected metabolic adaptations. Subgroup analyses were conducted only when each subgroup included at least 4 studies. Funnel plots and Egger’s regression test were used to assess publication bias. Potential publication bias was considered present when the funnel plot showed an asymmetric distribution or when Egger’s test yielded a *p-*value < 0.05. Following the approach adopted in a previous exercise meta-analysis (19, 26), leave-one-out sensitivity analyses were conducted by sequentially excluding each effect size and each primary study. The three-level model was re-estimated after each exclusion to determine whether any individual effect size or study exerted a disproportionate influence on the pooled estimates or heterogeneity. Changes in the pooled Hedges’ *g*, 95% confidence interval, and *I*^2^ were recorded.

## 3 Results

### 3.1 Literature search results

**FIGURE 1:**
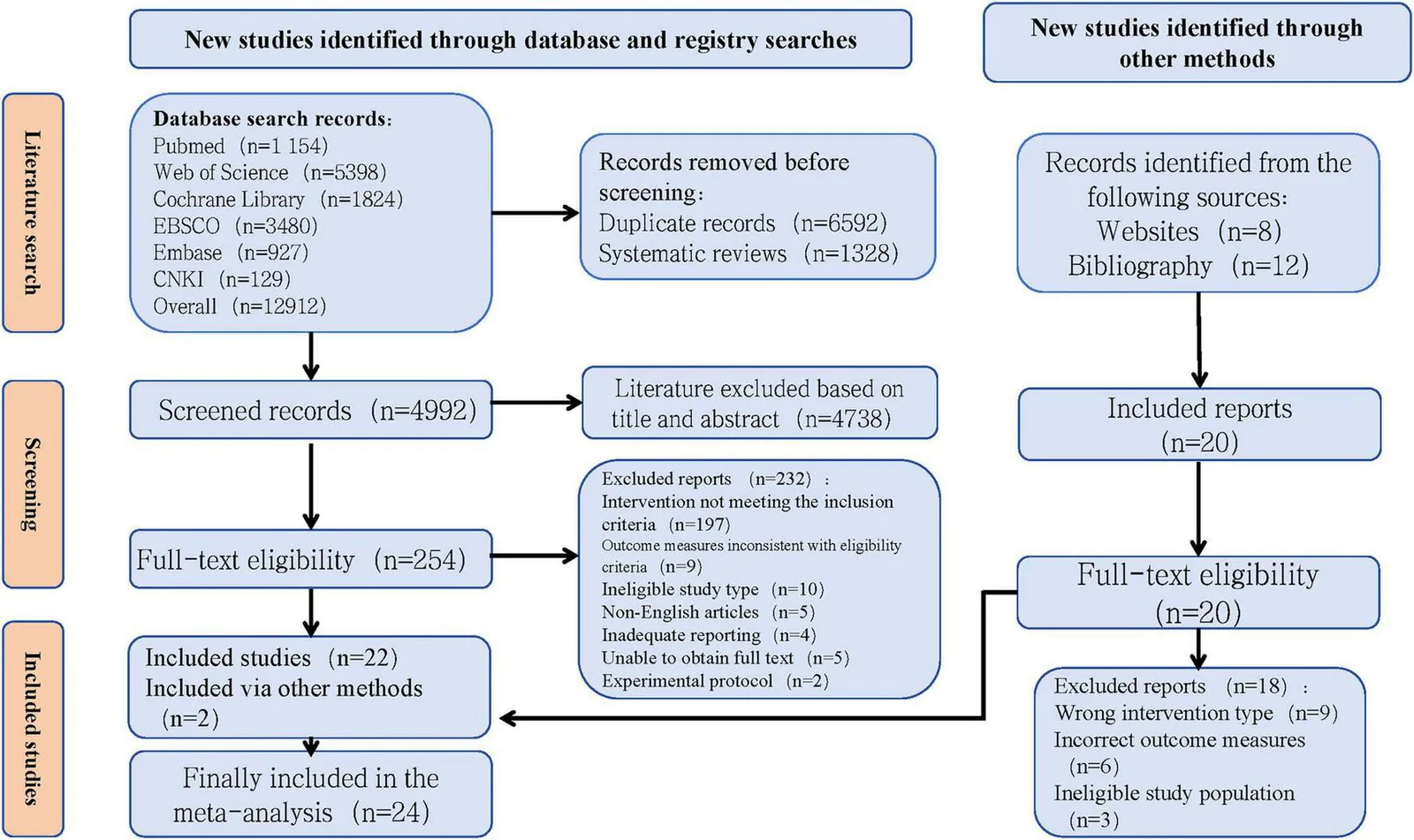
Flowchart of literature screening.

A total of 12,912 records were initially retrieved from Web of Science, PubMed, Cochrane Library, EBSCO, Embase, and CNKI. Two researchers screened titles, abstracts, and full texts according to PRISMA inclusion/exclusion criteria, ultimately including 24 studies. The literature screening flowchart is presented in Figure 1.

### 3.2 Characteristics of included studies

The characteristics of the included studies are summarized in Table 1. This study finally included 24 randomized controlled trials meeting inclusion/exclusion criteria (27–50), involving 2,578 participants (1,312 in exercise intervention groups and 1,266 in control groups). Publication years ranged from 2011 to 2026, including 3 Chinese studies (27–29) and 21 English studies (30–50). Participants ranged from 12.1 to 67.6 years old, predominantly middle-aged and elderly. Exercise intervention modalities included HIIT (6 studies) (29, 31, 33, 39, 43, 49, 50), MICT (7 studies) (27, 30, 38, 45–47, 50), RT (4 studies) (34, 36, 45, 48), and combined exercise (10 studies) (28, 32, 35, 37, 38, 40–44). Exercise equipment included treadmills (3 studies) (31, 45, 47), elliptical machines (1 study) (30), stationary bikes (8 studies) (29, 33, 39, 40, 43, 45, 49, 50), yoga training (1 study) (42), Nordic walking (1 study) (38), and combined walking/jogging/calisthenics (2 studies) (27, 29). Intervention durations ranged from 6 to 52 weeks (mostly 8 and 12 weeks), with individual session durations of 30–60 min and most studies using 3 sessions per week (Table 1).

**TABLE 1:** Basic characteristics of included studies.

First author	Year of publication	Group	Age (y)	Gender (male/female)	BMI	Training mode	Training duration	Frequency (wk)	Period	Outcome indicators
Abdelbasset al. (50)	2020	HIIT (*n* = 16)	54.4 ± 5.8	M 10/F 6	36.3 ± 4.5	Ergometer	40 Min	3 times	8 weeks	BMI, LDL-C, TG, HDL-C, ALT, VAT, HbA1c, TC
Abdelbasset al. (50)	2020	MICT (*n* = 15)	54.9 ± 4.7	M 8 /F 7	36.7 ± 3.4	Ergometer	40 min	3 times	8 weeks	
Abdelbasset al. (50)	2020	Control group (*n* = 16)	55.2 ± 4.3	M 9 /F 7	35.9 ± 5.3	Without exercise intervention	–	–	8 weeks	
Abdelbasset al. (49)	2019	HIIT (*n* = 16)	54.4 ± 5.8	M 10/F 6	36.3 ± 4.5	Ergometer	40 min	3 times	8 weeks	BMI, LDL-C, TG, HDL-C, ALT, VAT, HbA1c, TC
Abdelbasset al. (49)	2019	Control group (*n* = 16)	55.2 ± 4.3	M 9/F 7	35.9 ± 5.3	Without exercise intervention	–	–	8 weeks	
Al Ozairi et al. (48)	2023	RT (*n* = 64)	59.4 ± 8.5	M 23/F 23	30.56 ± 5.15	RT	30 min	3 times	32 weeks	BMI, LDL-C, TG, HDL-C, HbA1c, TC
Al Ozairi et al. (48)	2023	Control group (*n* = 56)	60.9 ± 10.3	M 23/F 23	31.84 ± 5.70	Without exercise intervention	–	–	32 weeks	
Al-Jiffri et al. (47)	2013	MICT (*n* = 50)	35.5 ± 8.7	M 24/F 26	32.11 ± 3.54	Treadmill	40 min	3 times	12 weeks	BMI, ALT, AST, GGT
		Control group (*n* = 50)	35.5 ± 9.1	M 25/F 25	32.37 ± 3.92	Without exercise intervention	–	–	12 weeks	
Arase et al. (46)	2013	MICT (*n* = 20)	64.5 ± 9.2	M 15/F 5	24.5 ± 2.6	AE	40 min	3 times	48 weeks	BMI, ALT, AST, FPG, HbA1c, TC
Arase et al. (46)	2013	Control group (*n* = 20)	64.5 ± 9.4	M 15/F 5	24.6 ± 2.8	AE	40 min	3 times	48 weeks	
Bacchi et al. (45)	2013	MICT (*n* = 14)	55.6 ± 2.0	M 10/F 4	30.5 ± 1.0	Treadmill/ergometer	60 min	3 times	16 weeks	BMI, TG, HDL-C, ALT, VAT, HbA1c, TC
Bacchi et al. (45)	2013	RT (*n* = 17)	56.0 ± 1.9	M 12/F 5	28.8 ± 1.1	RT	60 min	3 times	16 weeks	
Bacchi et al. (45)	2013	Control group	55.8 ± 2.1	M 9 / F 5	29.7 ± 1.2	Without exercise intervention	–	–	16 weeks	
Zhao et al. (28)	2025	CE (*n* = 40)	52.5 ± 8.6	M 27/F 13	25.52 ± 2.32	AE/RT	60 min	3 times	12 weeks	BMI, LDL-C, TG, HFC, HDL-C, ALT, AST, FPG, HbA1c
Zhao et al. (28)	2025	Control group(*n* = 40)	52.1 ± 9.6	M 27/F 13	25.86 ± 2.26	Without exercise intervention	60 min	3 times	12 weeks	
Balducci et al. (44)	2015	CE (*n* = 303)	58.5 ± 8.6	M 124/F 179	23.8 ± 2.7 kg/m^2^	AE/RT	40 min	2 times	48 weeks	BMI, HDL-C, ALT, AST, FLI, GGT
Balducci et al. (44)	2015	Control group (*n* = 303)	58.8 ± 8.5	M 131/F 172	24.1 ± 3.1 kg/m^2^	Without exercise intervention	–	–	48 weeks	
Banitaleb et al. (43)	2019	HIIT (*n* = 14)	55.3 ± 5.9	M 7/F 7	29.27 ± 3.00	Ergometer	30 min	3 times	10 weeks	BMI, ALT, AST, FLI, HbA1c, GGT
Banitaleb et al. (43)	2019	CE (*n* = 14)	54.1 ± 5.4	M 7/F 7	28.68 ± 4.34	Treadmill/resistance training	50 min	3 times	10 weeks	
Banitaleb et al. (43)	2019	Control group (*n* = 14)	55.7 ± 6.4	M 7/F 7	30.12 ± 3.52	Without exercise intervention	–	–	10 weeks	
Bayat et al. (42)	2026	CE (*n* = 18)	49.6 ± 3.6	M 9/F 9	31.06 ± 2.84	Cyclic Yoga	45 min	3 times	8 weeks	BMI, LDL-C, TG, HDL-C, ALT, AST, HbA1c, TC
Bayat et al. (42)	2026	Control group(*n* = 19)	50.3 ± 4.5	M 9/F 9	31.65 ± 4.25	Without exercise intervention	–	–	8 weeks	
Bianco et al. (41)	2023	CE (*n* = 58)	55.1 ± 7.3	M 29/F 29	30.20 ± 4.28 kg/m^2^	AE/RT	60 min	3 times	12 weeks	BMI, LDL-C, TG, HDL-C, ALT, AST, HbA1c, TC, GGT
Bianco et al. (41)	2023	Control group (*n* = 58)	56.3 ± 6.5	M 29/F 29	32.37 ± 5.17 kg/m^2^	Without exercise intervention	–	–	12 weeks	
Björnsdottir et al. (40)	2025	CE (*n* = 38)	55.7 ± 7.0	M 22/F 16	37.6 ± 5.8	Ergometer	30 min	3 times	8 weeks	BMI, LDL-C, TG, HFC, HDL-C, ALT, AST, HbA1c, TC
Björnsdottir et al. (40)	2025	Control group (*n* = 38)	55.5 ± 6.9	M 21 /F 17	37.4 ± 5.7	Without exercise intervention	–	–	8 weeks	
Cassidy et al. (39)	2016	HIIT (*n* = 12)	61.4 ± 9.2	M 10/F 2	31 ± 5	Ergometer	30 min	3 times	12 weeks	BMI, TG, HFC, ALT, AST, HbA1c, TC
Cassidy et al. (39)	2016	Control group (*n* = 11)	59.6 ± 9.7	M 8/F 3	32 ± 6	Without exercise intervention	–	–	12 weeks	
Cheng et al. (38)	2017	MICT (*n* = 29)	59.2 ± 4.4	M 6/F 23	27.3 ± 3.6	Nordic Walking	60 min	3 times	32 weeks	BMI, TG, HFC, ALT, AST, HbA1c, GGT
Cheng et al. (38)	2017	CE (*n* = 57)	60.0 ± 3.8	M 13/F 44	26.5 ± 2.8	Nordic walking	60 min	3 times	32 weeks	
Cheng et al. (38)	2017	Control group (*n* = 29)	60.8 ± 3.4	M 7/F 22	27.1 ± 2.9	Without exercise intervention	–	–	32 weeks	
Wang et al. (29)	2019	HIIT (*n* = 56)	52.1 ± 8.9	M 30/F 26	25.4 ± 2.3	Treadmill/cycling	40 min	5 times	12 weeks	BMI, LDL-C, TG, HDL-C, ALT, AST, FPG, HbA1c, TC, GGT
Wang et al. (29)	2019	Control group(*n* = 56)	52.6 ± 8.7	M 36/F 20	25.8 ± 2.5	Without exercise intervention	–	–	12 weeks	
Deng et al. (37)	2017	CE (*n* = 36)	63.7 ± 10.7	M 28/F 8	24.1 ± 2.6	AE	40 min	3 times	52 weeks	BMI, ALT, AST, HbA1c, TC
Deng et al. (37)	2017	Control group(*n* = 36)	64.1 ± 11.2	M 26/F 10	23.9 ± 2.4	AE	40 min	3 times	52 weeks	
Wei et al. (27)	2018	MICT (*n* = 70)	58.9 ± 9.8	M 38/F 32	28.75 ± 1.28	Brisk walking /Jogging	35 min	3 times	12 weeks	BMI, TG, ALT, AST, TC, GGT
Wei et al. (27)	2018	Control group (*n* = 70)	58.2 ± 9.3	M 40/F 30	28.52 ± 1.24	Without exercise intervention	–	–	12 weeks	
Freer et al. (36)	2022	RT (*n* = 19)	67.6 ± 5.2	M 10/F 6	31.5 ± 3.4	RT	45 min	3 times	48 weeks	BMI, TG, HFC, GGT
Freer et al. (36)	2022	Control group (*n* = 17)	66.9 ± 5.3	M 6/F 7	32.5 ± 3.8	Stretching exercise	45 min	3 times	48 weeks	
Grønbæk et al. (35)	2012	CE (*n* = 117)	12.1 ± 1.3	M 51/F 66	28.0 ± 3.6	AE	60 min	4 times	10 weeks	BMI, LDL-C, TG, HFC, HDL-C, TC, GGT
Grønbæk et al. (35)	2012	Control group (*n* = 117)	12.0 ± 1.2	M 49 /F 68	27.8 ± 3.5	Without exercise intervention	–	–	10 weeks	
Hallsworth et al. (34)	2011	RT (*n* = 11)	52 ± 13.3	M 23/F 18	32.3 ± 4.9	RT	60 min	3 times	8 weeks	BMI, TG, HFC, ALT, HbA1c, TC
Hallsworth et al. (34)	2011	Control group (*n* = 8)	62 ± 7.4	M 23/F 21	32.3 ± 4.8	Without exercise intervention	–	–	8 weeks	
Hallsworth et al. (33)	2015	HIIT (*N* = 12)	54 ± 10	M 7/F 5	31 ± 4	Ergometer	35 min	3 times	12 weeks	BMI, TG, HFC, ALT, AST, HbA1c, TC, GGT
Hallsworth et al. (33)	2015	Control group (*N* = 11)	52 ± 12	M 6/F 5	31 ± 5	Without exercise intervention	–	–	12 weeks	
Haxhi et al. (32)	2024	CE (*n* = 133)	61.7 ± 9.5	M 80/F 53	29.5 ± 4.9	AE/RT	40 min	3 times	48 weeks	BMI, TG, HFC, ALT, AST, HbA1c, TC, GGT
Haxhi et al. (32)	2024	Control group (*n* = 134)	62.7 ± 10.0	M 82/F 52	30.1 ± 5.6	Without exercise intervention	–	–	48 weeks	
Marcinko et al. (31)	2015	HIIT (*n* = 18)	51.7 ± 6.5	M 21/F 19	21.3 ± 2.6	Treadmill	30 min	3 times	6 weeks	BMI, HFC, ALT, AST, HbA1c
Marcinko et al. (31)	2015	Control group (*n* = 16)	52.3 ± 7.3	M 21/F 10	26.7 ± 3.6	Without exercise intervention	–	–	6 weeks	
Rabøl et al. (30)	2011	MICT (*n* = 12)	24.6 ± 5.2	M 6/F 6	24.3 ± 2.1	Elliptical	45 min	3 times	8 weeks	BMI, HFC, FPG
Rabøl et al. (30)	2011	Control group (*n* = 16)	23.8 ± 3.6	M 8/F 8	26.3 ± 3.5	Without exercise intervention	–	–	8 weeks	

### 3.3 Risk of bias assessment

**FIGURE 2:**
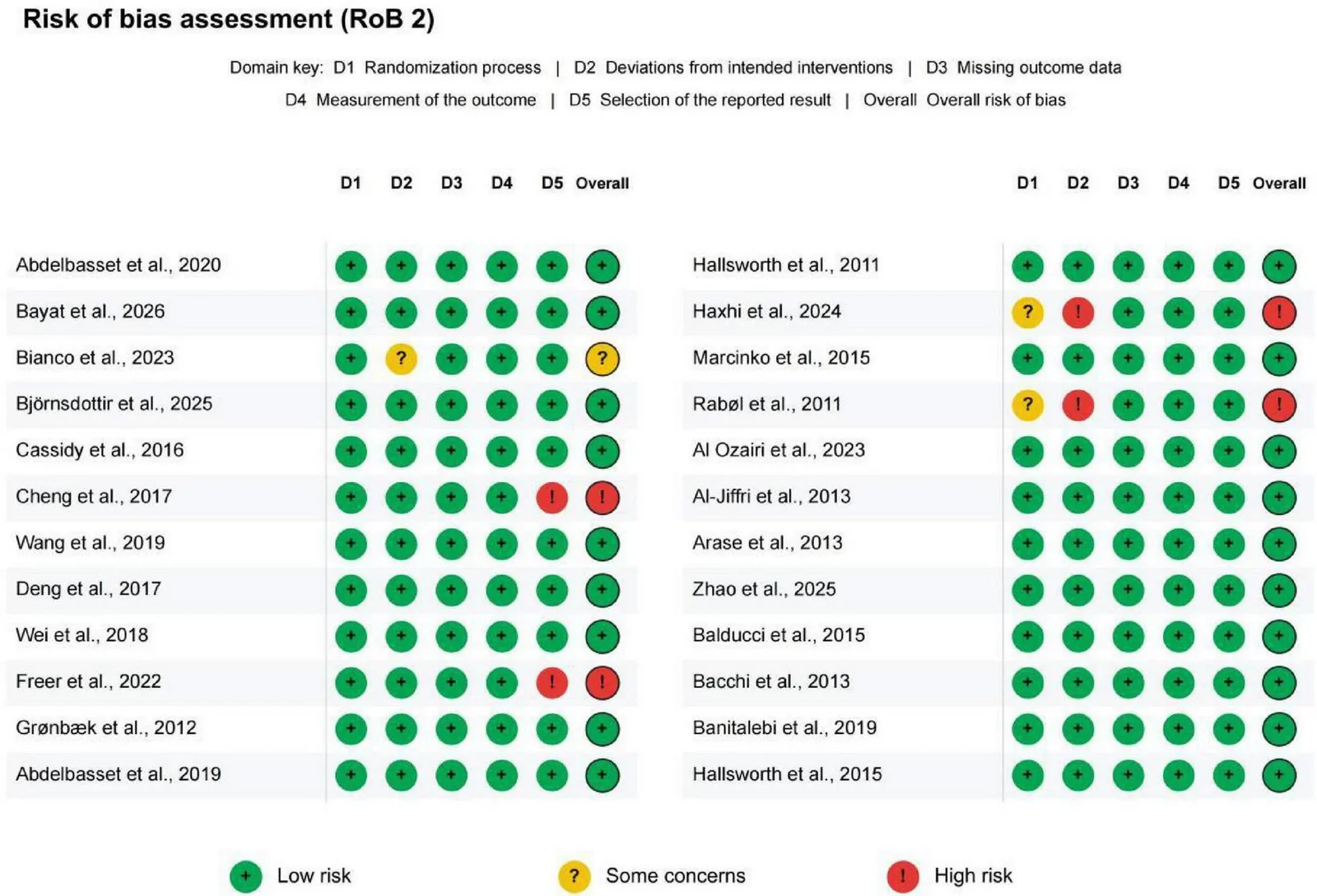
Risk of bias assessment of included studies.

The Cochrane Risk of Bias tool was used to assess risk of bias for all 24 included studies. Two studies did not report the randomization process (30, 32). Two studies had high risk in blinding implementation (30, 32), and one study had moderate risk (41). Two studies had high risk in selective reporting (36, 38). Overall, most studies showed no risk of bias, with only a few studies presenting some degree of bias risk (Figure 2).

### 3.4 Meta-analysis results

#### 3.4.1 Effects of exercise on glucose and lipid metabolism in T2DM patients with NAFLD

**FIGURE 3:**
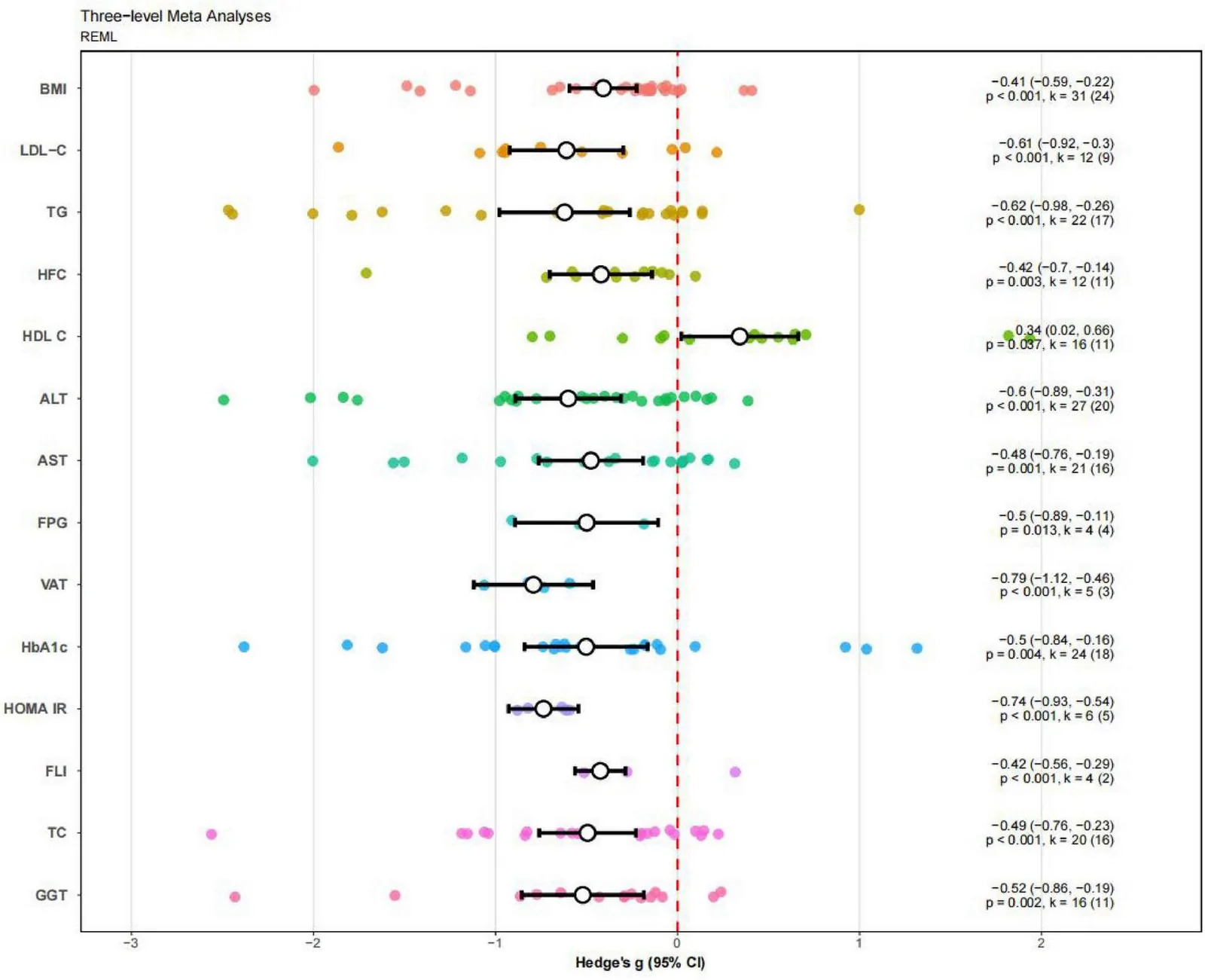
Effects of exercise on various metabolic indicators in patients with T2DM complicated with NAFLD.

The three-level meta-analysis showed that exercise intervention provided good improvement benefits for all metabolic indicators in T2DM patients with NAFLD. Exercise reduced BMI (Hedges’ *g* = −0.36, *p* < 0.001); improved glucose metabolism, reducing FPG (Hedges’ *g* = −0.42, *p* < 0.001), HbA1c (Hedges’ *g* = −0.52, *p* < 0.001), and HOMA-IR (Hedges’ *g* = −0.48, *p* < 0.001); improved lipid metabolism and fat distribution, reducing LDL-C (Hedges’ *g* = −0.36, *p* = 0.001), TG (Hedges’ *g* = −0.45, *p* < 0.001), TC (Hedges’ *g* = −0.31, *p* = 0.004), VAT (Hedges’ g = −0.49, *p* < 0.001), HFC (Hedges’ *g* = −0.42, *p* = 0.003), and FLI (Hedges’ *g* = −0.56, *p* < 0.001), while increasing HDL-C (Hedges’ *g* = 0.28, *p* = 0.009); and improved liver function, reducing ALT (Hedges’ *g* = −0.41, *p* < 0.001), AST (Hedges’ *g* = −0.38, *p* < 0.001), and GGT (Hedges’ *g* = −0.40, *p* < 0.001) (Figure 3).

#### 3.4.2 Subgroup analysis: effects of MICT

**FIGURE 4:**
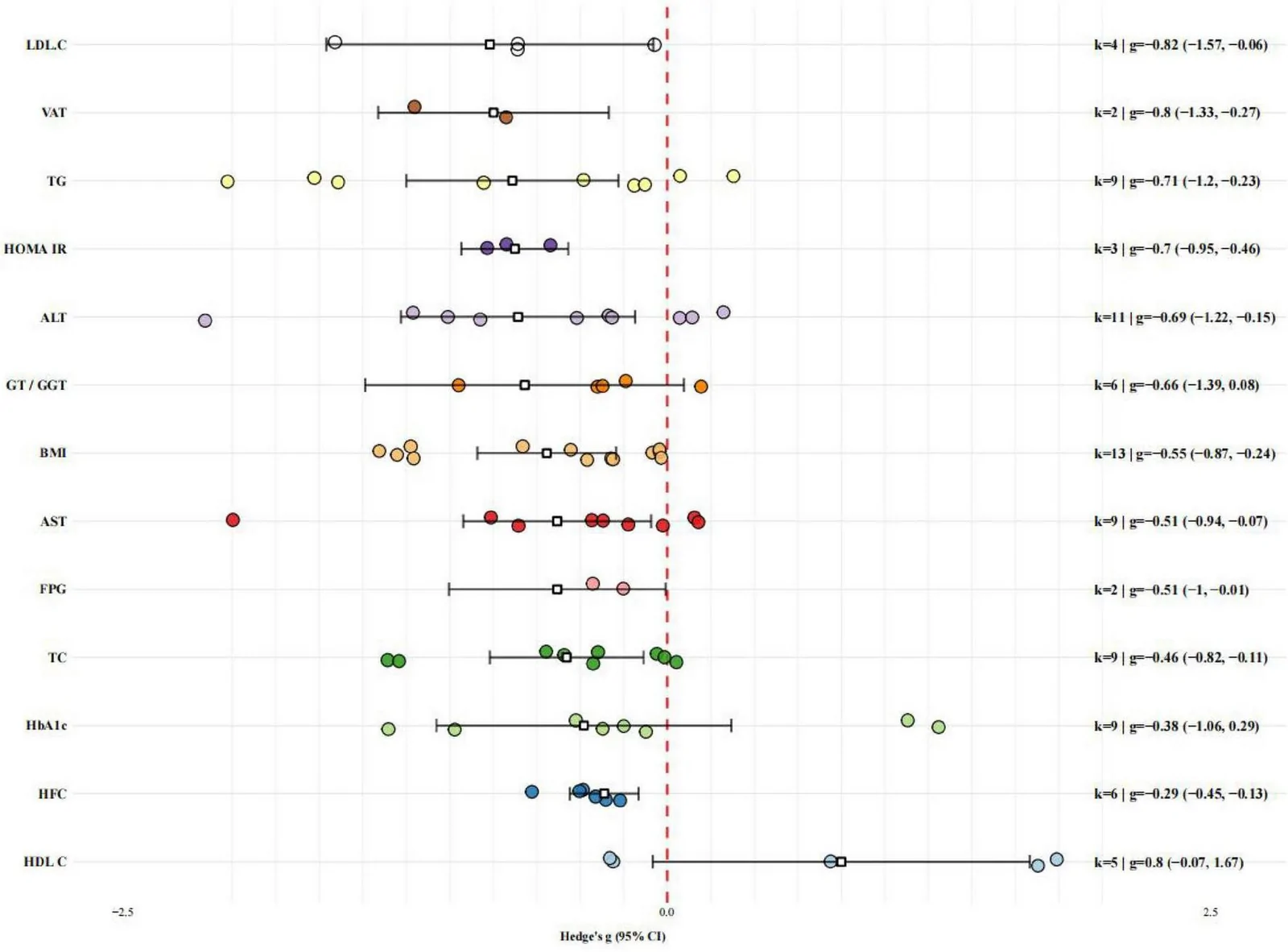
Effects of MICT on glucose and lipid metabolism in patients with T2DM complicated with NAFLD.

In the a priori subgroup analysis, MICT was associated with reductions in BMI (Hedges’ *g* = −0.55, *p* < 0.001), FPG (Hedges’ *g* = −0.51, *p* = 0.048), HOMA-IR (Hedges’ *g* = −0.70, *p* < 0.001), LDL-C (Hedges’ *g* = −0.82, *p* = 0.034), TG (Hedges’ *g* = −0.71, *p* = 0.004), TC (Hedges’ *g* = −0.46, *p* = 0.011), VAT (Hedges’ *g* = −0.80, *p* = 0.004), and HFC (Hedges’ *g* = −0.29, *p* = 0.001) (Figure 4). These findings suggest that MICT may be particularly relevant for patients requiring sustained improvements in fasting glucose, lipid profile, and visceral or hepatic fat accumulation.

#### 3.4.3 Subgroup analysis: effects of HIIT

**FIGURE 5:**
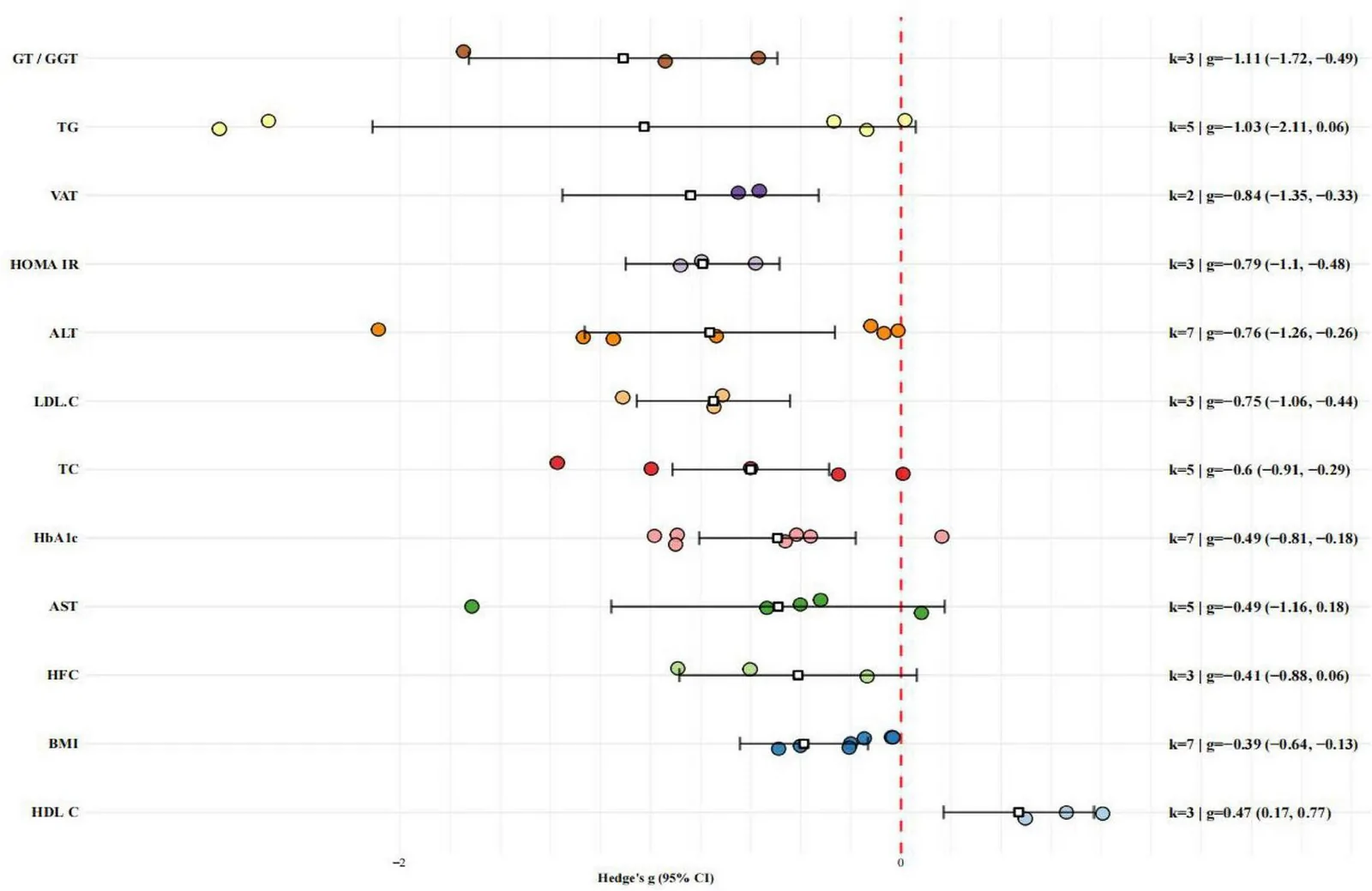
Effects of HIIT on glucose and lipid metabolism in patients with T2DM complicated with NAFLD.

In the HIIT subgroup, exercise reduced BMI (Hedges’ *g* = −0.39, *p* = 0.002), GGT (Hedges’ *g* = −1.11, *p* < 0.001), TG (Hedges’ *g* = −1.03, *p* = 0.026), VAT (Hedges’ *g* = −0.84, *p* = 0.001), ALT (Hedges’ *g* = −0.76, *p* = 0.003), LDL-C (Hedges’ *g* = −0.75, *p* < 0.001), TC (Hedges’ *g* = −0.60, *p* < 0.001), HOMA-IR (Hedges’ *g* = −0.79, *p* < 0.001), and HbA1c (Hedges’ *g* = −0.49, *p* = 0.002) (Figure 5). The larger effects observed for several outcomes may reflect the higher intensity stimulus of HIIT, but tolerability, safety, adherence, and baseline cardiovascular risk should be considered before clinical implementation.

#### 3.4.4 Subgroup analysis: effects of RT

**FIGURE 6:**
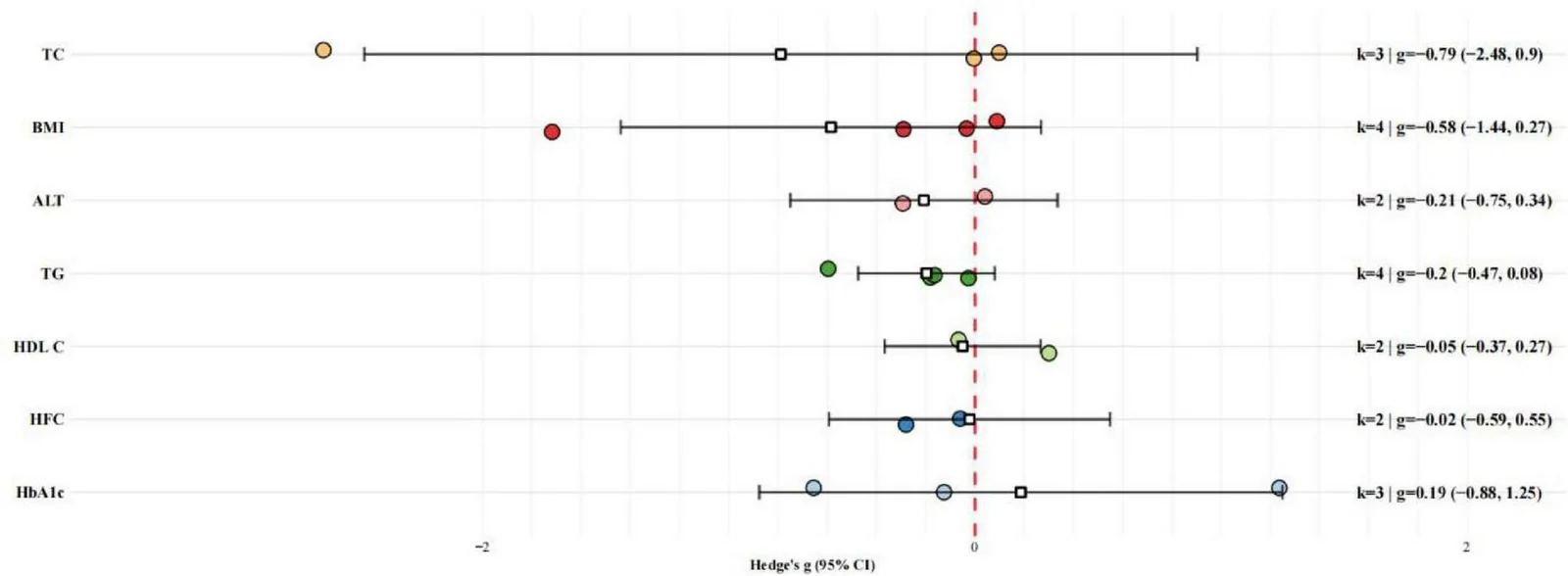
Effects of resistance training (RT) on glycemic and lipid metabolism in patients with T2DM combined with NAFLD.

In the RT subgroup, the pooled effects on BMI, HbA1c, TC, TG, HDL-C, HFC, and ALT did not reach statistical significance (*p* > 0.05) (Figure 6). This finding should not be interpreted as evidence that RT is ineffective. Only a small number of RT studies were available, sample sizes were limited, and intervention protocols varied; therefore, the non-significant results may reflect insufficient statistical power and clinical heterogeneity.

#### 3.4.5 Subgroup analysis: effects of combined exercise

**FIGURE 7:**
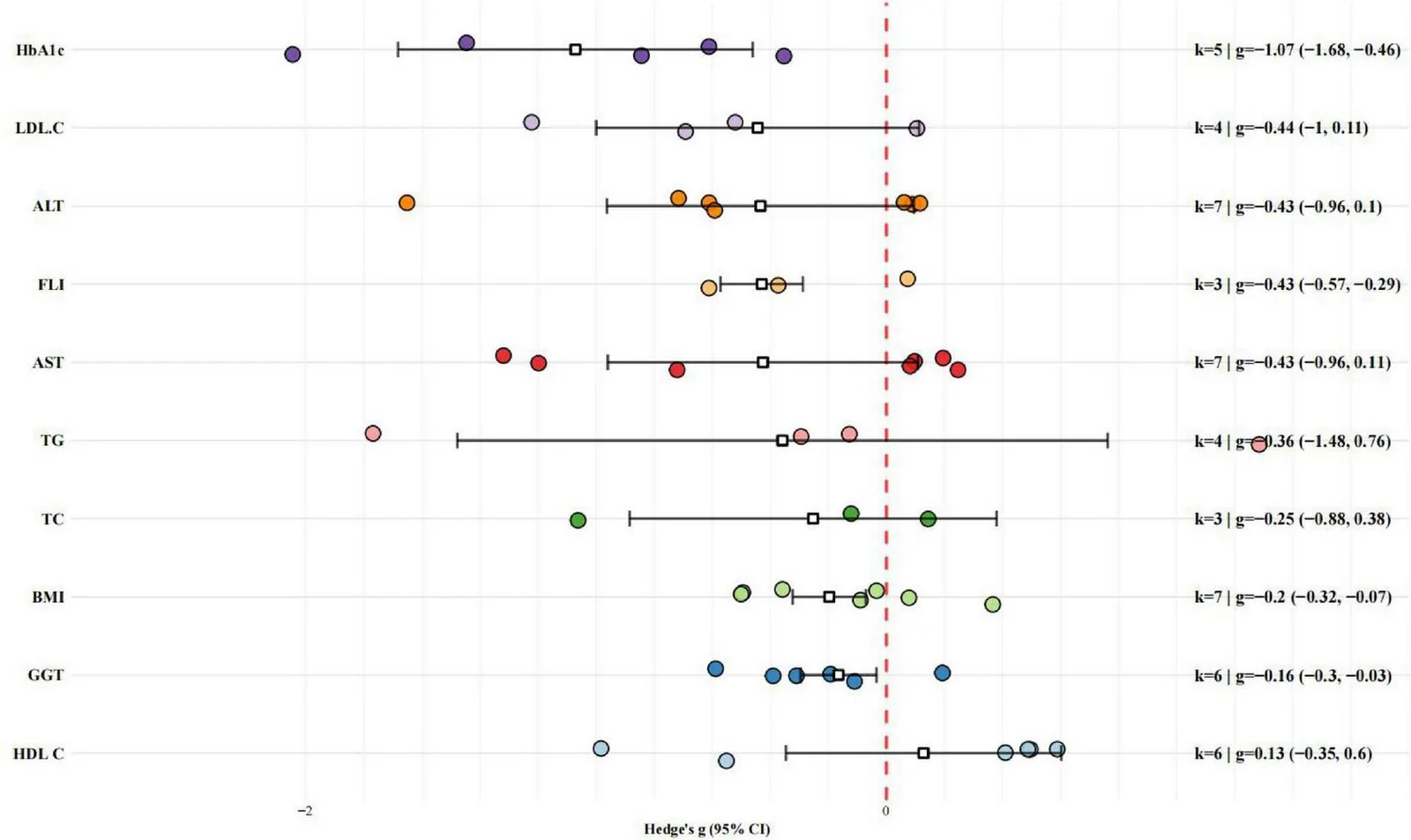
Effects of combined exercise on glycemic and lipid metabolism in T2DM with NAFLD.

For combined exercise, pooled estimates suggested reductions in BMI (Hedges’ *g* = −0.20, *p* = 0.007), HbA1c (Hedges’ *g* = −1.07, *p* = 0.001), FLI (Hedges’ *g* = −0.43, *p* = 0.026), and GGT (Hedges’ *g* = −0.16, *p* = 0.023) (Figure 7). Because combined programs differed in the proportion, sequence, and dose of aerobic and resistance components, these results should be interpreted in relation to total weekly exercise volume and adherence.

#### 3.4.6 Comparative analysis of aerobic exercise effects on glycolipid metabolism

**FIGURE 8:**
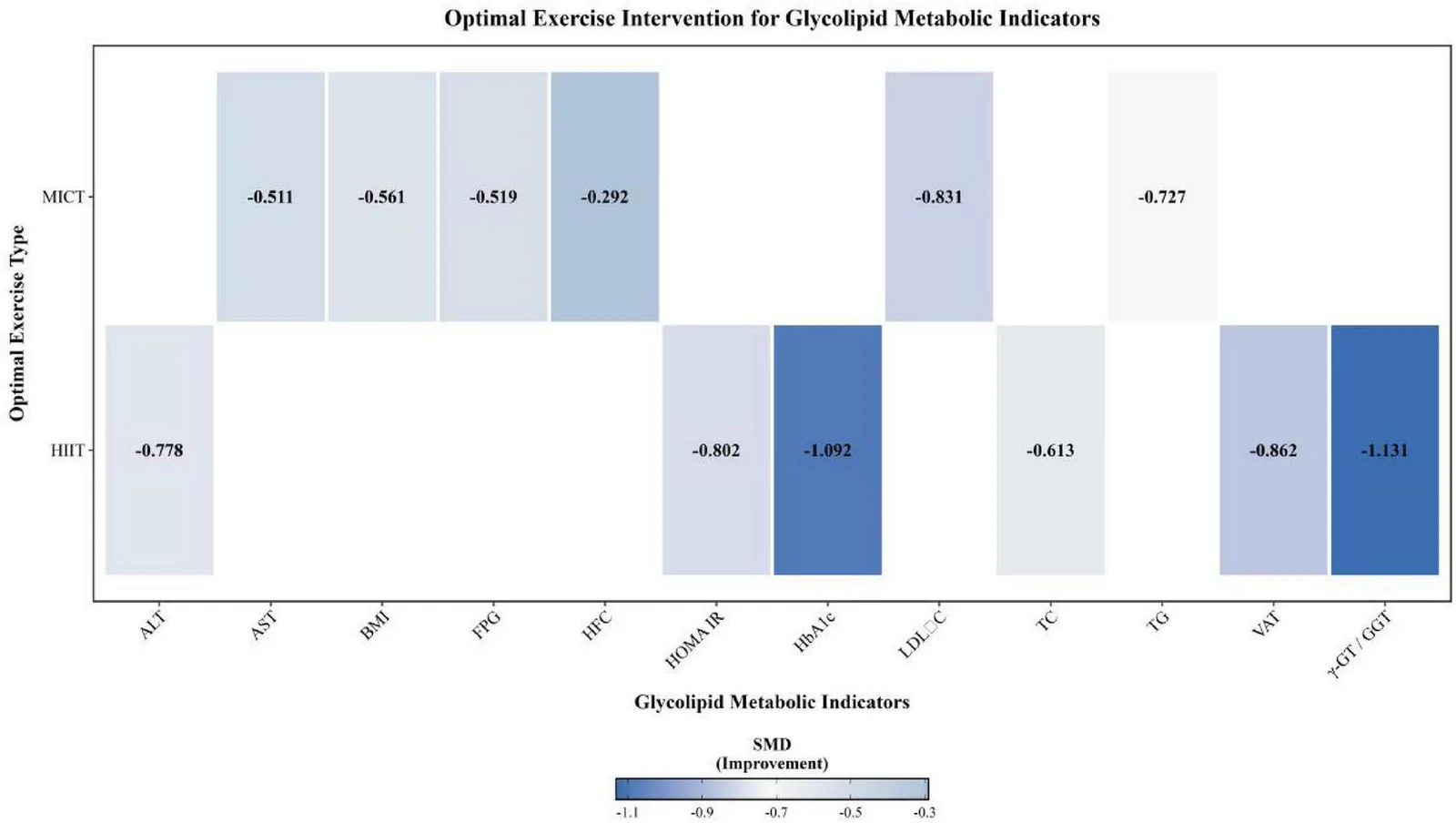
Comparative analysis of aerobic exercise effects on glycolipid metabolism.

Heatmap results showed that MICT and HIIT each have advantages in improving glucose and lipid metabolism indicators in T2DM patients with NAFLD: MICT is more suitable for improving body morphology (BMI: Hedges’ *g* = −0.519), controlling fasting blood glucose (FPG: Hedges’ *g* = −0.519), reducing HFC (Hedges’ *g* = −0.292), LDL-C (Hedges’ g = −0.831), and VAT (Hedges’ *g* = −0.727); HIIT is the optimal intervention for improving glucose metabolism (HOMA-IR: Hedges’ *g* = −0.802; HbA1c: Hedges’ *g* = −1.092), lipid metabolism (TC: Hedges’ *g* = −0.613; TG: Hedges’ *g* = −0.862), and liver function (GGT: Hedges’ *g* = −1.131; ALT: Hedges’ *g* = −0.778) (Figure 8).

#### 3.4.7 Sensitivity analysis and publication bias

**FIGURE 9:**
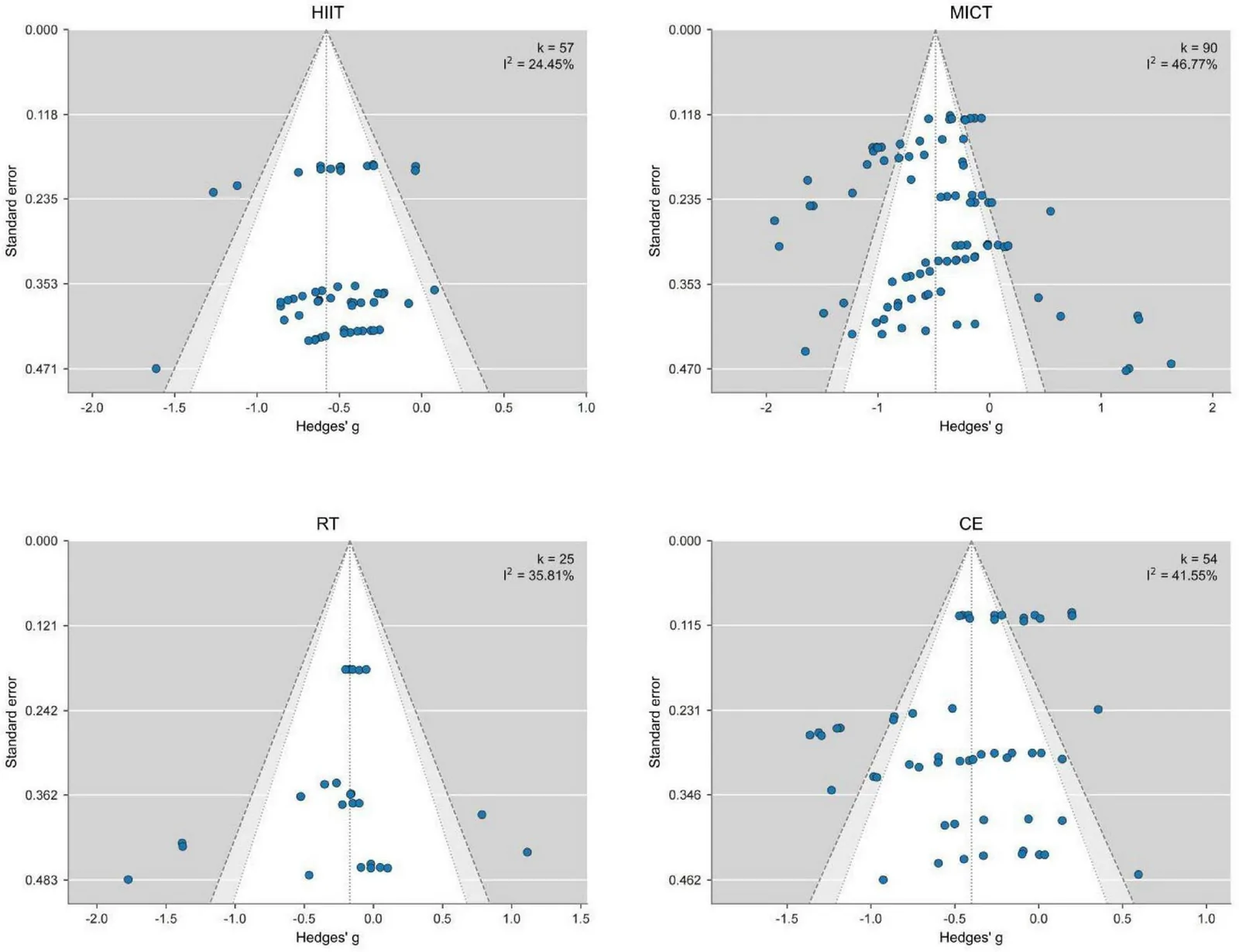
Summary funnel plots of the effects of different exercise modes on glycolipid metabolism in patients with T2DM combined with NAFLD.

**FIGURE 10:**
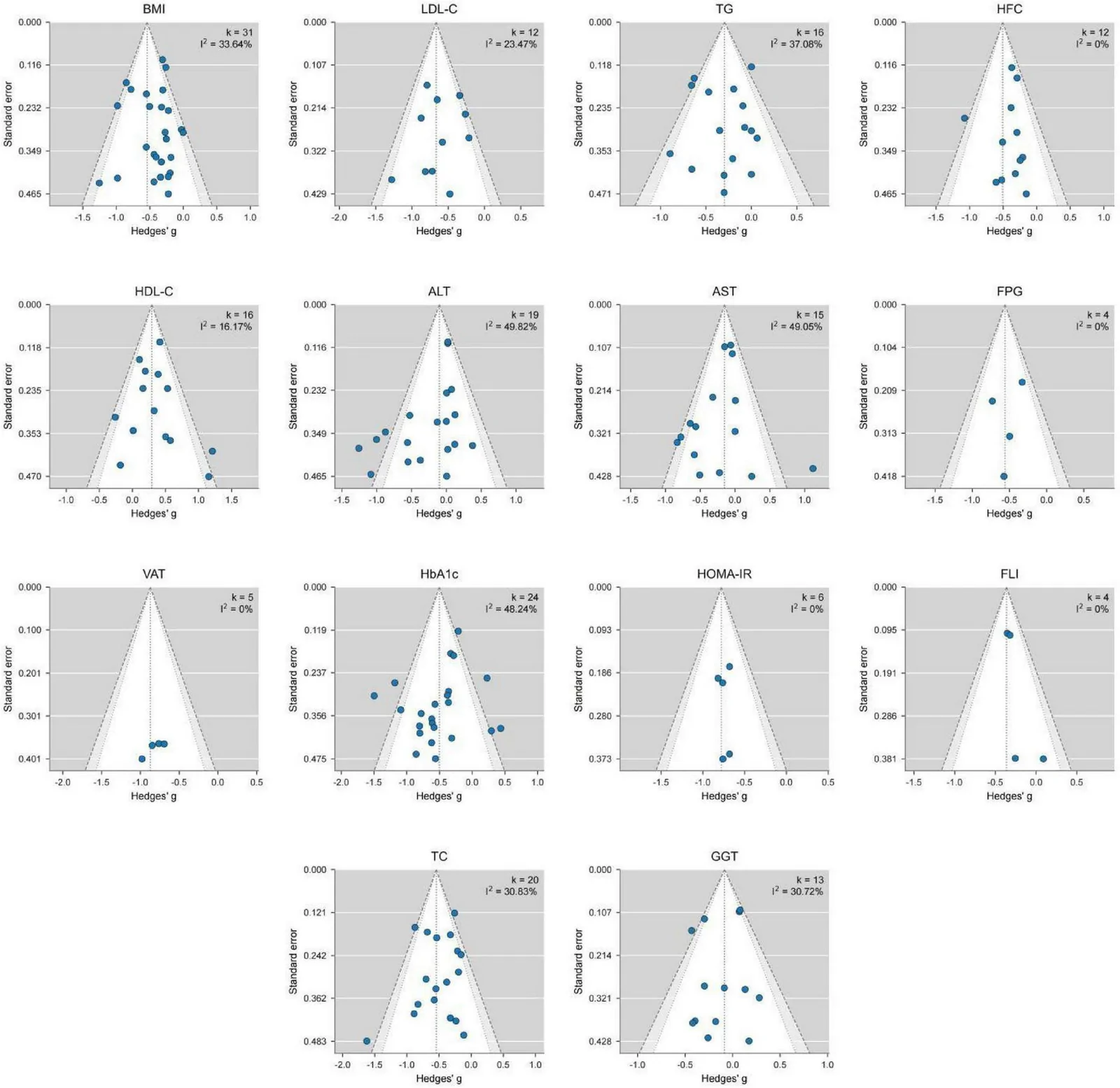
Summary funnel plots of the effects of exercise on glycolipid metabolism in T2DM patients with NAFLD.

Funnel plots were used to visually assess the distribution of study effect sizes across exercise modality subgroups (Figure 9) and different glucose and lipid metabolic outcomes (Figure 10). The HIIT, MICT, RT, and CE subgroup analyses showed relatively low to moderate heterogeneity (*I*^2^ = 24.45–46.77%), and the effect sizes were approximately symmetrically distributed on both sides of the funnels. Egger’s regression tests were not statistically significant (*t* = −2.897 to −1.043, all *p* ≥ 0.05), suggesting no clear evidence of substantial publication bias. In the leave-one-out sensitivity analyses, the test statistic *r* varied only from −0.128 to −0.111 after the sequential exclusion of each individual effect size or primary study, indicating that the current meta-analysis results were robust and reliable.

Publication bias was further assessed for the 14 included outcomes. Nine outcomes showed low heterogeneity (*I*^2^ = 0–38.83%), and Egger’s regression tests were not statistically significant (*t* = −1.241 to −0.069, all *p* ≥ 0.05). TG, ALT, AST, HbA1c, and GGT exhibited substantial heterogeneity (*I*^2^ ≥ 50%). After excluding studies contributing to the high heterogeneity, heterogeneity was reduced for TG (*k* = 16, *I*^2^ = 23.47%), ALT (*k* = 19, *I*^2^ = 49.82%), AST (*k* = 15, *I*^2^ = 49.05%), HbA1c (*k* = 24, *I*^2^ = 48.24%), and GGT (*k* = 13, *I*^2^ = 30.72%), with no material changes in the overall pooled effects. The sensitivity-analysis statistic *r* ranged from −0.187 to −0.029, further supporting the robustness of the findings (Figure 10).

#### 3.4.8 Heterogeneity sources and meta-regression analysis

Meta-regression suggested that medication status (β : -81.152 to 6.846), exercise frequency (β : −27.662 to 2.16), exercise duration (β : −0.726 to 1.247), and intervention length (β : −0.76 to −0.007) were potential study-level moderators of heterogeneity in TG, ALT, AST, and GGT (Table 2).

**TABLE 2:** Results of meta-regression analysis for indicators with high heterogeneity.

Indicator	Influencing factors	β	95% CI-L	95% CI-U	*p*	*R* ^2^
ALT	Medication use	3.691	−7.784	15.165	< 0.001	15.20%
	Frequency	−0.66	−8.263	6.943	0.038	1.85%
	Duration	−0.361	−0.901	0.178	0.049	2.81%
	Weeks	−0.116	−0.298	0.066	0.211	2.21%
AST	Medication use	3.003	−7.621	13.626	< 0.001	18.40%
	Frequency	2.16	−4.043	8.363	0.029	4.62%
	Duration	−0.465	−0.917	−0.013	0.044	13.75%
	Weeks	−0.07	−0.23	0.09	0.047	1.50%
GGT	Medication use	6.846	−5.216	18.908	< 0.001	22.60%
	Frequency	1.026	−6.684	8.736	0.015	8.74%
	Duration	−0.726	−1.178	−0.274	0.002	38.57%
	Weeks	−0.007	−0.182	0.169	0.049	0.50%
TG	Medication use	−81.152	−128.707	−33.597	< 0.001	33.79%
	Frequency	−27.662	−63.847	8.522	0.041	5.79%
	Duration	1.247	−1.553	4.047	0.035	4.20%
	Weeks	−0.76	−1.62	0.099	0.048	9.02%

## 4 Discussion

This study employed three-level meta-analysis to systematically include 24 randomized controlled trials, comprehensively evaluating the intervention effects of different exercise modalities on glucose and lipid metabolism and liver function indicators in T2DM patients with NAFLD, and exploring the core sources of heterogeneity. Results confirmed that aerobic exercise (HIIT, MICT) is an effective approach for improving metabolic disorders.

### 4.1 Effects of aerobic exercise on glucose metabolism in T2DM patients with NAFLD

The present three-level meta-analysis showed that aerobic exercise significantly reduced FPG (Hedges’ *g* = −0.42, *p* < 0.001), HbA1c (Hedges’ *g* = −0.52, *p* < 0.001), and HOMA-IR (Hedges’ *g* = −0.48, *p* < 0.001). These findings indicate improvements in fasting glucose regulation, long-term glycemic control, and insulin resistance. They are consistent with previous meta-analyses showing that exercise improves HbA1c and HOMA-IR in patients with T2DM and NAFLD, and reduces HbA1c in the broader T2DM population (25–27).

The prespecified subgroup analyses further showed that MICT reduced FPG (Hedges’ *g* = −0.51, *p* = 0.048) and HOMA-IR (Hedges’ *g* = −0.70, *p* < 0.001), whereas HIIT reduced HbA1c (Hedges’ *g* = −0.49, *p* = 0.002) and HOMA-IR (Hedges’ *g* = −0.79, *p* < 0.001) (Figures 4, 5, 8). A previous meta-analysis restricted to patients with T2DM and NAFLD similarly reported improvements in HbA1c and HOMA-IR following both HIIT and MICT (25). Broader evidence also supports the beneficial effects of HIIT on insulin resistance, fasting glucose, and HbA1c (28). However, direct HIIT-versus-MICT meta-analyses have generally found no significant difference between the two modalities in glycemic outcomes (29). Therefore, the patterns observed in Figure 8 should be interpreted as exploratory modality-specific signals rather than definitive evidence of the superiority of HIIT or MICT. The shared glycemic benefits of HIIT and MICT are biologically plausible. Skeletal muscle contraction stimulates glucose uptake through insulin-independent and insulin-dependent pathways involving AMPK activation (30, 31). Repeated aerobic training may further increase mitochondrial biogenesis, oxidative capacity, glucose-transporter expression, and skeletal-muscle insulin sensitivity (30–32). HIIT produces a greater acute metabolic perturbation and can induce substantial mitochondrial remodeling despite a relatively low training volume (33). Exercise intensity is also positively associated with excess post-exercise oxygen consumption, although this response represents only a modest proportion of total exercise energy expenditure and should not be regarded as direct evidence of improved insulin resistance (34). MICT provides a sustained moderate-intensity stimulus that may facilitate the accumulation of a consistent weekly exercise volume, which could contribute to its favorable effect on FPG.

The improvement in glycemic control may not require substantial weight loss. A previous meta-analysis found that exercise reduced HbA1c by approximately 0.66% without a significant between-group difference in body mass (27). This evidence supports the biological plausibility of weight-independent glycemic benefits. Nevertheless, the present meta-analysis did not formally test whether changes in BMI mediated the improvements in FPG, HbA1c, or HOMA-IR. Medication use, dietary co-interventions, baseline glycemic control, exercise dose, and adherence may also influence treatment responses. Consequently, modality-specific findings should be interpreted cautiously and applied according to individual cardiovascular risk, exercise tolerance, and metabolic profile.

### 4.2 Effects of aerobic exercise on lipid metabolism in T2DM patients with NAFLD

This study confirmed that aerobic exercise can significantly improve lipid metabolism disorders in T2DM patients with NAFLD, effectively reducing TG, TC, and LDL-C levels while increasing HDL-C levels and reducing ectopic fat deposits such as VAT and HFC (Figures 4, 5). The regulatory effects of aerobic exercise on lipid metabolism are multi-target and multi-pathway (51). HIIT showed more prominent improvement effects on TG and TC. HIIT can rapidly activate the body’s energy metabolism pathways by enhancing anaerobic and aerobic metabolic capacity (52), significantly increasing fat oxidation and decomposition rates, while inducing mitochondrial biogenesis in skeletal muscle, increasing mitochondrial number and function, and enhancing the body’s lipid utilization efficiency (53). Short et al. (54) believed that HIIT can continue to elevate metabolic rate and extend lipid oxidation time after exercise cessation, more efficiently reducing blood TG content and improving hepatic lipid synthesis and metabolism imbalance. MICT showed unique advantages in reducing VAT and lowering LDL-C, which may be the preferred exercise modality for improving fat distribution abnormalities in T2DM patients with NAFLD. MICT can promote systemic blood circulation and fat mobilization through sustained aerobic exercise stimulation, with significant effects on visceral fat reduction (55). Vissers et al. (55) believed that visceral adipose tissue is more responsive to sustained aerobic exercise. Continuous energy expenditure can effectively reduce visceral fat accumulation and decrease VAT, while reduction of visceral fat can further improve intra-abdominal insulin resistance and alleviate lipid metabolism disorders. Mann et al. (56) found that MICT can regulate hepatic lipoprotein metabolism, inhibit LDL-C synthesis and secretion, and enhance clearance efficiency. As the core atherogenic lipid indicator, LDL-C reduction can significantly reduce cardiovascular complication risks.

### 4.3 Effects of aerobic exercise on liver function in T2DM patients with NAFLD

Aerobic exercise can effectively reduce ALT, AST, and GGT levels, with HIIT showing particularly prominent improvement effects on GGT, while MICT has more advantages in stable regulation of ALT and AST. Liver function damage is a typical clinical feature of T2DM patients with NAFLD (57). ALT and AST, as core markers of hepatocyte injury, reflect hepatocyte degeneration and necrosis. GGT is closely related to hepatic lipid metabolism and detoxification function, and its high expression often indicates NAFLD progression and increased intrahepatic steatosis severity (58). This study confirmed that aerobic exercise can specifically improve these liver function indicators, suggesting it has definite hepatoprotective effects on T2DM patients with NAFLD. The improvement effects of aerobic exercise on liver function are achieved by improving glucose and lipid metabolism disorders and alleviating intrahepatic steatosis and inflammatory response.

Due to insulin resistance and lipid metabolism disorders in T2DM patients with NAFLD, large amounts of lipids abnormally deposit in the liver, forming hepatic steatosis, and excessive hepatic fat accumulation triggers oxidative stress reactions in hepatocytes (59). Li et al. (60) believed that aerobic exercise can increase the body’s antioxidant capacity, enhance the activity of various antioxidant enzymes, and clear excessive reactive oxygen species in hepatocytes, thereby reducing hepatocyte degeneration and necrosis, reducing ALT and AST release, and achieving liver function indicator improvement. MacInnis et al. (61) found that aerobic exercise can improve hepatic lipid metabolism and cell membrane structure, inhibit GGT synthesis and release, while enhancing hepatic detoxification function, further alleviating intrahepatic oxidative stress and inflammatory response, forming a positive cycle of hepatoprotection. Additionally, this study found that aerobic exercise improvement of liver function is significantly correlated with improvement of glucose and lipid metabolism and fat distribution (Figure 3). This suggests that correction of glucose and lipid metabolism disorders and reduction of intrahepatic fat deposition are prerequisites for liver function improvement, while liver function improvement further enhances the liver’s capacity for glucose and lipid metabolism and alleviates insulin resistance, forming a virtuous cycle of glucose and lipid metabolism and liver function improvement (62).

### 4.4 Analysis of heterogeneity sources and influencing factors

Based on the three-level meta-regression, medication status, exercise frequency, session duration, and intervention length were associated with between-study heterogeneity in TG, ALT, AST, and GGT. Medication status accounted for 15.20–33.79% of the between-study heterogeneity across these outcomes. However, these study-level associations should not be interpreted as evidence that medication use was the primary or causal source of heterogeneity.

Medication-related heterogeneity is biologically plausible. Previous research has examined the combined effects of metformin and exercise on metabolic and mitochondrial outcomes (63). Statin-associated muscle symptoms may also affect patientsologicaise tolerance or adherence (64). Because the included studies were randomized controlled trials, exercise effects were estimated from between-group comparisons. When medication regimens were maintained or applied similarly in the intervention and control groups, randomization and comparable co-interventions may have reduced medication-related confounding within individual trials. However, medication class, dose, treatment duration, and regimen changes were inconsistently reported, and the study-level medication coding could not distinguish the effects of specific drugs. Therefore, residual medication-related confounding cannot be excluded, and the meta-regression findings should be interpreted cautiously.

Exercise prescription parameters (exercise frequency, individual session duration, intervention weeks) are another important cause of heterogeneity. Richter et al. (15) believed that regular exercise three or more times per week can continuously activate the AMPK signaling pathway, maintain the body’s stable regulatory capacity for glucose and lipid metabolism, and continuously reduce intrahepatic fat deposition and hepatocyte inflammation. MacInnis et al. (61) believed that appropriate individual session duration can ensure total exercise load, fully achieve fat mobilization and hepatic fatty acid oxidation effects, reduce exercise compliance and even cause exercise injuries, thereby affecting intervention effects. Palacios et al. (65) believed that sustained exercise for 10 weeks or more can achieve stable reduction in TG levels. Although short-term exercise can produce transient blood lipid improvement, it is difficult to form lasting effects, which may also explain why TG improvement effects are not significant in some short-term intervention studies.

## 5 Future research directions

Future studies should conduct adequately powered, multicenter randomized controlled trials that directly compare HIIT, MICT, RT, and combined exercise using standardized exercise protocols and consistent outcome measures. Medication use, dietary intake, baseline glycemic control, diabetes duration, BMI, NAFLD severity, and exercise adherence should be prospectively recorded and appropriately controlled to reduce residual confounding. Longer intervention and follow-up periods are also required to evaluate the durability and safety of exercise-induced benefits. Further dose-response and individual participant-level analyses may help identify the optimal exercise modality, intensity, frequency, and duration for different patient profiles, thereby supporting more precise and personalized exercise prescriptions.

## 6 Conclusion

This three-level meta-analysis suggests that exercise improves glycemic regulation, lipid metabolism, and liver function in patients with T2DM and NAFLD. HIIT and MICT may provide complementary metabolic benefits, while combined exercise was associated with improvements in BMI, HbA1c, FLI, and GGT. Evidence regarding resistance training (RT) remains inconclusive because of the limited number of studies and small sample sizes; therefore, nonsignificant findings should not be interpreted as evidence of no benefit. Current evidence does not establish the superiority of any single exercise modality, and exercise prescriptions should be individualized according to patientstiontabolic targets, medication use, and exercise tolerance.

## 7 Limitations

Several limitations should be acknowledged. First, the included studies inconsistently reported clinical characteristics such as participants’ age, sex, diabetes duration, and baseline glycemic control. Medication use, dietary interventions, and other co-interventions may also have confounded the findings; however, the available data were insufficient to support comprehensive subgroup or meta-regression analyses. In addition, the limited number of studies and small sample sizes in the resistance training subgroup may have reduced statistical power. Therefore, nonsignificant findings should not be interpreted as evidence of no benefit. Finally, most studies had relatively short intervention and follow-up periods, warranting caution when interpreting the long-term effectiveness and safety of exercise interventions.

## Data Availability

The original contributions presented in the study are included in the article/Supplementary material, further inquiries can be directed to the corresponding author.
