# Supplementary material for: Effects of exercise intervention on glucose and lipid metabolism in patients with type 2 diabetes mellitus complicated by non-alcoholic fatty liver disease: a three-level meta-analysis
Source: Front Nutr. 2026 Aug 11;13:1907058. doi: 10.3389/fnut.2026.1907058 (PMC13503576; doi:10.3389/fnut.2026.1907058)
Supplement: Supplementary file 2 [file Table_3.pdf]

# Supplementary Table S3. GRADE evidence profile for major outcomes

Population: patients with type 2 diabetes mellitus and non-alcoholic fatty liver disease. Intervention: structured exercise. Comparator: usual care, maintenance of habitual activity, or no exercise intervention.

| Outcome | Effect sizes / studies / participants | Risk of bias | Inconsistency | Indirectness | Imprecision | Publication bias | Pooled effect | Certainty | Reason for certainty judgment |
|---|---|---|---|---|---|---|---|---|---|
| **Body mass index (BMI)** | 31 effects / 24 RCTs<br>N = 2,257 | Not serious[a] | Not serious<br>($I^2$ = 33.64%) | Not serious | Not serious | Not detected[e] | g = -0.41<br>(95% CI -0.59 to -0.22) | ⊕⊕⊕⊕<br>High | No serious concern was identified across the five GRADE domains. |
| **Glycated hemoglobin (HbA1c)** | 24 effects / 18 RCTs<br>N = 1,213 | Not serious[a] | Serious[b] | Not serious | Not serious | Not detected[e] | g = -0.50<br>(95% CI -0.84 to -0.16) | ⊕⊕⊕○<br>Moderate | Downgraded one level because substantial heterogeneity was present in the primary analysis. |
| **Fasting plasma glucose (FPG)** | 4 effects / 4 RCTs<br>N = 256 | Not serious[a] | Not serious<br>($I^2$ = 0%) | Not serious | Serious[c] | Not assessable[e] | g = -0.50<br>(95% CI -0.89 to -0.11) | ⊕⊕⊕○<br>Moderate | Downgraded one level for imprecision because the total sample was limited and the CI ranged from a small to a large benefit. |
| **Homeostatic model assessment of insulin resistance (HOMA-IR)** | 6 effects / 5 RCTs<br>N = 431 | Not serious[a] | Not serious<br>($I^2$ = 0%) | Not serious | Not serious | Not assessable[e] | g = -0.74<br>(95% CI -0.93 to -0.54) | ⊕⊕⊕⊕<br>High | The estimate was precise, consistent, and directly relevant; publication bias could not be formally assessed because fewer than 10 studies were available. |
| **Low-density lipoprotein cholesterol (LDL-C)** | 12 effects / 9 RCTs<br>N = 774 | Not serious[a] | Not serious<br>($I^2$ = 23.47%) | Not serious | Not serious | Not assessable[e] | g = -0.61<br>(95% CI -0.92 to -0.30) | ⊕⊕⊕⊕<br>High | No serious concern was identified; formal assessment of publication bias was limited by the number of studies. |
| **Triglycerides (TG)** | 22 effects / 17 RCTs<br>N = 1,389 | Not serious[a] | Serious[b] | Not serious | Not serious | Not detected[e] | g = -0.62<br>(95% CI -0.98 to -0.26) | ⊕⊕⊕○<br>Moderate | Downgraded one level for substantial heterogeneity in the primary analysis; sensitivity analysis did not materially change the pooled effect. |
| **High-density lipoprotein cholesterol (HDL-C)** | 16 effects / 11 RCTs<br>N = 1,384 | Not serious[a] | Not serious<br>($I^2$ = 16.17%) | Not serious | Serious[c] | Not detected[e] | g = 0.34<br>(95% CI 0.02 to 0.66) | ⊕⊕⊕○<br>Moderate | Downgraded one level because the CI included effects ranging from trivial to moderate benefit. |
| **Total cholesterol (TC)** | 20 effects / 16 RCTs<br>N = 1,322 | Not serious[a] | Not serious<br>($I^2$ = 30.83%) | Not serious | Not serious | Not detected[e] | g = -0.49<br>(95% CI -0.76 to -0.23) | ⊕⊕⊕⊕<br>High | No serious concern was identified across the five GRADE domains. |
| **Alanine aminotransferase (ALT)** | 27 effects / 20 RCTs<br>N = 1,897 | Not serious[a] | Serious[b] | Not serious | Not serious | Not detected[e] | g = -0.60<br>(95% CI -0.89 to -0.31) | ⊕⊕⊕○<br>Moderate | Downgraded one level for substantial heterogeneity in the primary analysis; the direction of effect remained stable in sensitivity analysis. |
| **Aspartate aminotransferase (AST)** | 21 effects / 16 RCTs<br>N = 1,752 | Not serious[a] | Serious[b] | Not serious | Not serious | Not detected[e] | g = -0.48<br>(95% CI -0.76 to -0.19) | ⊕⊕⊕○<br>Moderate | Downgraded one level for substantial heterogeneity in the primary analysis; sensitivity analysis supported robustness. |
| **Gamma-glutamyl transferase (GGT)** | 16 effects / 11 RCTs<br>N = 1,613 | Not serious[a] | Serious[b] | Not serious | Not serious | Not detected[e] | g = -0.52<br>(95% CI -0.86 to -0.19) | ⊕⊕⊕○<br>Moderate | Downgraded one level for substantial heterogeneity in the primary analysis; the pooled direction remained stable after sensitivity analysis. |

| Outcome | Effect sizes / studies / participants | Risk of bias | Inconsistency | Indirectness | Imprecision | Publication bias | Pooled effect | Certainty | Reason for certainty judgment |
|---|---|---|---|---|---|---|---|---|---|
| **Hepatic fat content (HFC)** | 12 effects / 11 RCTs<br>N = 826 | Not serious[a] | Not serious<br>($I^2$ = 0%) | Not serious | Not serious | Not detected[e] | g = -0.42<br>(95% CI -0.70 to -0.14) | ⊕⊕⊕⊕<br>High | The pooled estimate was consistent and directly relevant, with no serious concern identified across GRADE domains. |
| **Visceral adipose tissue (VAT)** | 5 effects / 3 RCTs<br>N = 125 | Not serious[a] | Not serious<br>($I^2$ = 0%) | Not serious | Serious[c] | Not assessable[e] | g = -0.79<br>(95% CI -1.12 to -0.46) | ⊕⊕⊕○<br>Moderate | Downgraded one level for imprecision because only three studies with 125 participants contributed to the estimate. |
| **Fatty liver index (FLI)** | 4 effects / 2 RCTs<br>N = 605 | Not serious[a] | Not serious<br>($I^2$ = 0%) | Serious[d] | Not serious | Not assessable[e] | g = -0.42<br>(95% CI -0.56 to -0.29) | ⊕⊕⊕○<br>Moderate | Downgraded one level for indirectness because FLI is a surrogate index rather than a direct imaging or histological measure of liver fat. |

Abbreviations: CI, confidence interval; GRADE, Grading of Recommendations Assessment, Development and Evaluation; RCT, randomized controlled trial.

All outcomes started at high certainty because the evidence was derived from randomized controlled trials. The number of participants represents analyzed participants in the effect-size dataset; shared control groups were counted once within each outcome.

[a] Risk of bias was not downgraded because most trials were judged as low risk or with some concerns, outcomes were predominantly objective laboratory, anthropometric, or imaging measures, and leave-one-out analyses did not materially change the pooled estimates. The inability to blind participants and personnel in exercise trials remains an inherent limitation.

[b] Downgraded one level for inconsistency because the primary analysis showed substantial heterogeneity ($I^2 \geq 50\%$). Although sensitivity analyses reduced $I^2$ below 50% without materially changing the pooled effect, the downgrade was retained because the reduced estimates followed study exclusions and clinical heterogeneity remained.

[c] Downgraded one level for imprecision because the total sample was small or the 95% CI included effects ranging from trivial/small to moderate/large benefit.

[d] Downgraded one level for indirectness because FLI is a surrogate algorithm rather than a direct imaging or histological assessment of hepatic steatosis.

[e] Funnel plots and Egger's regression showed no clear evidence of publication bias when sufficient studies were available. Formal assessment was considered unreliable when fewer than 10 studies contributed to an outcome; this limitation was noted but was not automatically used as a downgrading criterion.

Effect interpretation: Hedges' g values of approximately 0.2, 0.5, and 0.8 were considered small, moderate, and large standardized effects, respectively. Negative values favor exercise for all listed outcomes except HDL-C, for which positive values favor exercise.
