# Supplementary material for: Effects of exercise intervention on glucose and lipid metabolism in patients with type 2 diabetes mellitus complicated by non-alcoholic fatty liver disease: a three-level meta-analysis
Source: Front Nutr. 2026 Aug 11;13:1907058. doi: 10.3389/fnut.2026.1907058 (PMC13503576; doi:10.3389/fnut.2026.1907058)
Supplement: Supplementary file 3 [file Table_1.docx]

April 1, 2026

Dear Editor-in-Chief,

We are submitting our manuscript titled "Effects of Exercise Intervention on Glucose and Lipid Metabolism in Patients with Type 2 Diabetes Mellitus Complicated by Non-Alcoholic Fatty Liver Disease: A Three-Level Meta-Analysis" for consideration for publication in your esteemed journal.

This systematic review and meta-analysis addresses a critical clinical challenge: managing glucose and lipid metabolism disorders in patients with type 2 diabetes mellitus (T2DM) complicated by non-alcoholic fatty liver disease (NAFLD). Our study makes several important contributions to the field:

1. Novel Methodology: We employed a three-level meta-analysis approach, which allows for the simultaneous analysis of multiple effect sizes within studies, avoiding statistical power overestimation and providing more robust estimates of exercise intervention effects.

2. Comprehensive Evaluation: Our analysis included 24 randomized controlled trials with 2,578 participants, systematically evaluating the effects of different exercise modalities including high-intensity interval training (HIIT), moderate-intensity continuous training (MICT), resistance training (RT), and combined exercise on 14 glucose and lipid metabolism indicators.

3. Key Findings: We found that aerobic exercise (both HIIT and MICT) significantly improved metabolic parameters, with HIIT showing superior effects on HbA1c, GGT, and triglycerides, while MICT demonstrated advantages in controlling fasting blood glucose and LDL-C. Importantly, both modalities showed weight-independent benefits.

4. Clinical Implications: Our meta-regression analysis identified medication status and exercise prescription parameters (frequency, duration, and intervention length) as key moderating factors, providing evidence-based guidance for personalized exercise prescription in clinical practice.

5. This is the first systematic review to comprehensively compare the effects of different exercise modalities on T2DM patients with NAFLD using three-level meta-analysis, providing valuable insights for clinicians in developing precision exercise interventions.

The findings of this study are particularly relevant given the high comorbidity of T2DM and NAFLD (50-70% prevalence) and the limited effectiveness of pharmacological interventions in this population. We believe this work will be of significant interest to clinicians, researchers, and healthcare policymakers working in diabetes management, hepatology, and exercise medicine.

All authors have approved the manuscript and declare no conflicts of interest. This study was registered in PROSPERO (CRD420261327724) and conducted in accordance with PRISMA 2020 guidelines.

Thank you for considering our manuscript. We look forward to hearing from you.

Sincerely,

**Wang Xuan, Liu Wenshuo, Jin Zongqiang*, Niu Yanmei**

School of Sports Training, Tianjin University of Sport

Tianjin 301617, China

E-mail: zongqiangjin@126.com
